# Development and evaluation of a droplet digital PCR assay for the diagnosis of paucibacillary leprosy in skin biopsy specimens

**DOI:** 10.1371/journal.pntd.0007284

**Published:** 2019-03-18

**Authors:** Xiujun Cheng, Lele Sun, Qing Zhao, Zihao Mi, Gongqi Yu, Zhenzhen Wang, Yonghu Sun, Chuan Wang, Chunhua Man, Fanghui Fu, Hong Liu, Furen Zhang

**Affiliations:** 1 Shandong Provincial Institute of Dermatology and Venereology, Shandong Academy of Medical Sciences, Jinan, Shandong, China; 2 Shandong Provincial Key Laboratory for Dermatovenereology, Jinan, Shandong, China; 3 National Clinical Key Project of Dermatology and Venereology, Jinan, Shandong, China; 4 Shandong Provincial Hospital for Skin Diseases, Shandong University, Jinan, Shandong, China; 5 School of Medicine and Life Science, University of Jinan-Shandong Academy of Medical Sciences, Jinan, Shandong, China; Faculty of Science, Ain Shams University (ASU), EGYPT

## Abstract

**Background:**

The reduced amounts of *Mycobacterium leprae* (*M*. *leprae*) among paucibacillary (PB) patients reflect the need to further optimize methods for leprosy diagnosis. An increasing number of reports have shown that droplet digital polymerase chain reaction (ddPCR) is a promising tool for diagnosis of infectious disease among samples with low copy number. To date, no publications have investigated the utility of ddPCR in the detection of *M*. *leprae*. The aim of this study was to develop and evaluate a ddPCR assay for the diagnosis of PB leprosy.

**Methodology:**

The two most sensitive DNA targets for detection of *M*. *leprae* were selected from electronic databases for assessment of sensitivity and specificity by quantitative polymerase chain reaction (qPCR) and ddPCR. Control patients (n = 59) suffering from other dermatological diseases were used to define the cut-off of the duplex ddPCR assay. For comparative evaluation, qPCR and ddPCR assays were performed in 44 PB patients and 68 multibacillary (MB) patients.

**Principal findings:**

*M*. *leprae*-specific repetitive element (*RLEP*) and *groEL* (encoding the 65 kDa molecular chaperone GroEL) were used to develop the ddPCR assay by systematically analyzing specificity and sensitivity. Based on the defined cut-off value, the ddPCR assay showed greater sensitivity in detecting *M*. *leprae* DNA in PB patients compared with qPCR (79.5% vs 36.4%), while both assays have a 100% sensitivity in MB patients.

**Conclusions/Significance:**

We developed and evaluated a duplex ddPCR assay for leprosy diagnosis in skin biopsy samples from leprosy patients. While still costly, ddPCR might be a promising diagnostic tool for detection of PB leprosy.

## Author summary

Leprosy, or Hansen’s disease, is a chronic bacterial disease caused by *M*. *leprae*. Although it is curable and early treatment averts most disabilities, it remains an important global health concern. This is mainly due to delayed diagnosis. In leprosy, a reliable and early diagnostic tool, is still needed. In recent decades years, the quantitative PCR (qPCR) based on nucleic acid detection has been employed for leprosy diagnosis, which exhibited high sensitivity. The performance of qPCR assays, however, greatly varied in different studies, especially in the diagnosis of PB patients. ddPCR is a new and sensitive method used in the examination of pathogenic microorganism, showing considerable reliability and efficiency in other infectious diseases. To our knowledge, no publication reported the ddPCR assay for leprosy diagnosis. Herein, we developed and evaluated a ddPCR assay for detecting *M*. *leprae* in skin biopsy samples. Our results suggest that ddPCR specially targeting *RLEP* and *groEL* genes could be a promising tool to the detection of *M*. *leprae* in PB leprosy with a higher sensitivity than qPCR. This research provides a new molecular biology methods for leprosy diagnosis.

## Introduction

Leprosy, a chronic infectious disease caused by *M*. *leprae*, has a tropism for macrophages in skin and Schwann cells in peripheral nerves [1]. This disease is quite variable, affecting people in different ways according to their immune response. At one end of the spectrum, patients with a high level of immunity harbor a low number of bacilli and are termed PB patients. Patients with many bacilli are referred to as MB patients [2]. Despite its elimination as a global public health problem due to the widespread implementation of multidrug therapy, leprosy continues to mar the lives of the infected individuals [3]. In 2016, a total of 214,783 new patients of which 12,819 were detected with visible deformities, were reported in 143 countries among all World Health Organization regions filed, corresponding to a global new case detection rate of 2.9 per 100,000 population[4]. The principal consideration in measuring the reduction of leprosy burden has been the decrease the number of visible deformities among new patients [4]. Therefore, early diagnosis and prompt treatment remain key strategies for leprosy control [1,4].

Because the main diagnostic tools for leprosy involve bacillary counts with a limited sensitivity of 30% and histopathology showing a specific neural inflammation histopathologic changes, which require well-experienced clinicians, late diagnosis is frequently the case for many patients [2,3]. Although serological tests and IFN-γ releasing assays have also been used to detect *M*. *leprae* as potential diagnostic tools [5,6], PB patients are negative for phenolic glycolipid-1 and household contacts exhibit a similar pattern of IFN-γ secretion as PB patients [5–7]. In the past three decades, identification of *M*. *leprae* DNA has become popular through the development of PCR methods for leprosy diagnosis [4,7]. As 33%-83% of PB patients have been confirmed by PCR, this has greatly aided clinicians in identifying leprosy patients with negative bacilloscopic and inconclusive histopathological features [7]. For MB patients who have high bacillary loads are easily detected by PCR, and the sensitivity of qPCR is almost 100% [7].

The ddPCR, based on water-oil emulsion droplet technology, is a new PCR method for nucleic acid detection [8–11]. Several studies on ddPCR have shown its higher sensitivity and precision in molecular diagnostics for pathogens such as *hepatitis B virus* [8], *human immunodeficiency virus* (HIV) [9], *chlamydia trachomatis* [10] and *chromosomally integrated human herpes virus 6* [11]. To the best of our knowledge, no publications have reported on the clinical utility of the ddPCR assay for leprosy. Here, we developed a ddPCR assay for the diagnosis of leprosy in skin biopsy specimens and compared the diagnostic performance of ddPCR and qPCR on leprosy.

## Methods

### Ethics statement

The study was approved by the institutional review board (IRB) committee of the Shandong Provincial Institute of Dermatology and Venereology, Shandong Academy of Medical Science, China (IRB approval number: 2016-KYKT-29). We followed the Genetic Risk Prediction Studies guidelines [12] and written informed consent was obtained from each participant and all of whom were adult subjects.

### Patients and samples

A total of 112 leprosy patients (comprising 68 MB and 44 PB patients) and 59 non-leprosy patients from Shandong Provincial Hospital for Skin Diseases (Shandong, China) were collected and enrolled in this study. All patients were of Chinese descent. The confirmed diagnoses were based on systematic analysis and integration of patients’ medical history, clinical manifestations, slit skin smear staining, histological examinations.

We used *Mycobacterium marinum* (*M*. *marinum*) and *Mycobacterium tuberculosis* to evaluate the specificity of the assays. *M*. *marinum* was provided by Dr. Annemarie H. Meijer (Department of Molecular Cell Biology, Institute of Biology, Leiden University, Leiden, Netherlands) and eight DNA samples from sputum of patients infected by *Mycobacterium tuberculosis* were provided by Jinan infectious disease hospital.

### Genomic DNA extraction

DNA was extracted from skin biopsies and *M*. *marinum* using QIAamp DNA Mini Kits (Qiagen) according to the manufacturer’s instructions. Extracted DNA was measured with a NanoDrop 8000 spectrophotometer (Thermo Scientific) and then either used immediately or stored at -80°C.

### Primers and probes

Following the guidelines for reporting systematic reviews from PRISMA [13], we searched PubMed and EMBASE from their inception until March 25, 2018 to assure a comprehensive study. Six genes, including *RLEP*, 18 kDa heat shock protein (*HSP18*), antigen 85B (*Ag 85B*), superoxide dismutase A (*sodA*), 16S ribosomal Ribose Nucleic Acid (*16SrRNA*) and early secretory antigenic target (*esxA)*, have been used in Taqman qPCR previously [14–17]. For other 11 genes, their primers and probes were designed by Premier 3.0 based on the DNA sequences in previous studies [17–26]. The primers and probes of all 17 genes were summarized in S1 Table.

### Selection of two most sensitive target genes

Five DNA samples were chosen among 68 MB patients as representative to systematically evaluate the sensitivity of 17 genes. Briefly, the DNA was firstly normalized using ddPCR based on the target gene of *Ag85B*, which had shown as the most specific target gene in previous publications [14]. Then the DNA samples were diluted to 1,000 copies/ul, followed by increasing dilutions (1:10, 1:100, 1:200, 1:1,000, 1:2,000, 1:10,000, 1:20,000 and 1:100,000). Finally, the two most sensitive genes (*RLEP* and *groEL*) from 17 target genes were selected according to the highest dilutions that could be detected by qPCR and ddPCR (limit of detection (LOD)).

### qPCR

qPCR was performed in duplicate using the ABI Step One Plus real-time PCR system (Applied BioSystems). PCR reaction mixtures were 20 μL in volume and contained 10 μL of 2× TaqMan Gene Expression Master Mix (Applied BioSystems), 900 nM primers, 250 nM probes and 4 μL of extracted DNA. The qPCR condition was as follows: 50˚C for 2 min and 95˚C for 10 min, followed by 40 cycles of 15 s at 95˚C and 1 min at 60˚C. Fluorescent accumulation data were analyzed using the ABI StepOne Software Version 2.2.2 (Applied Biosystems). The threshold cycle (CT) values of < 37 was defined a positive result for the qPCR assay. After determination of the two most sensitive target genes (*RLEP* and *groEL*), the qPCR was performed in all samples enrolled in this study, which were considered as positive when three or four wells (*RLEP* and *groEL* in duplicate) have positive signals (CT< 37).

### ddPCR

The ddPCR was performed in duplicate using a QX200 Droplet Digital PCR system (Bio-Rad). Each assay mix was prepared in a final volume of 20 μL, containing 10 μL of 2× ddPCR Supermix for Probes (no dUTP; Bio-Rad), 900 nM primers, 250 nM probes and 4 μL of extracted DNA. The generation of droplets was performed by the QX200 Droplet Generator (Bio-Rad) according to the manufacturer’s protocols. PCR amplification was carried out on an Applied Biosystems Veriti 96-Well Thermal Cycler using the following PCR conditions: 95°C for 10 min followed by 40 cycles of 94°C for 30 s, 60°C for 1 min and a final extension step at 98°C for 5 min. The plate was stored at 16°C until droplets were analyzed by the QX200 Droplet Reader and QuantaSoft software version 1.7.4 (Bio-Rad). The ddPCR of *RLEP* and *groEL* genes was performed in all samples enrolled in this study, and the fluorescent signal events above the threshold line were evaluated. A positive well was defined if more than four fluorescent signal events were shown above the threshold line. The samples were determined as positive when the four test wells (*RLEP* and *groEL* in duplicate) showed at least three positive wells.

The detailed protocols regarding qPCR and ddPCR are available in protocols.io in the following: dx.doi.org/10.17504/protocols.io.v4ye8xw; dx.doi.org/10.17504/protocols.io.v4ze8x6.

### Statistical analysis

Data were statistically described in terms of range, mean ± standard deviation (SD), frequency (number of patients) and relative frequency (percentages). The statistical significance of the differences in sensitivities between ddPCR and qPCR were assessed by means of the kappa test and McNemar test. The differences of age between MB, PB and non-leprosy patients were assessed by ANOVA test, race and gender were assessed by Chi-square test. This manuscript followed the Standards for the Reporting of Diagnostic accuracy studies (STARD) (S1 File, S2 File, S3 File).

## Results

### Subjects

A total of 171 patients including 68 MB patients, 44 PB patients and 59 non-leprosy patients were enrolled in this study. All clinical characteristics of these 171 subjects are provided in Table 1. This study consisted of 109 males (63.7%) and 62 females (36.3%). The mean age of MB patients, PB patients and non-leprosy patients were 44.9 (range from 13 to 77), 45.7 (range from 19 to 80) and 44.8 (range from 18 to 78), respectively. 135 subjects (78.9%) were Chinese Han descent. There was no difference regarding the gender, age and ethnicity among these three groups (all P values > 0.05).

**Table 1:** Baseline characteristics of the study subjects that used in the qPCR and ddPCR assays.

Subject	Gender (no. (%))		P value	Age in years (mean±SD)	P value	Ethnicity (no. (%))		P Value
	Male	Female				Han	Other Minorities (Buyi, Miao, Yi, Chuang, Yao)	
**MB patients (N = 68)**	45 (66.2)	23 (33.8)	0.742	44.9±15.8	0.956	53 (77.9)	15 (22.1)	0.323
**PB patients (N = 44)**	26 (59.1)	18 (40.9)		45.7±18.1		32 (72.7)	12 (27.3)	
**Non-leprosy patients (N = 59)**	38 (64.4)	21 (35.6)		44.8±15.7		50 (84.7)	9 (15.3)	
**Total (N = 171)**	109 (63.7)	62 (36.3)		45.1±16.3		135 (78.9)	36 (21.1)	

### The selection of DNA sequences for the duplex ddPCR assay

Every primers and their related probes of all 17 genes were aligned with the *M*. *leprae* genome using Basic Local Alignment Search Tool software (NCBI).

**Fig 1:**
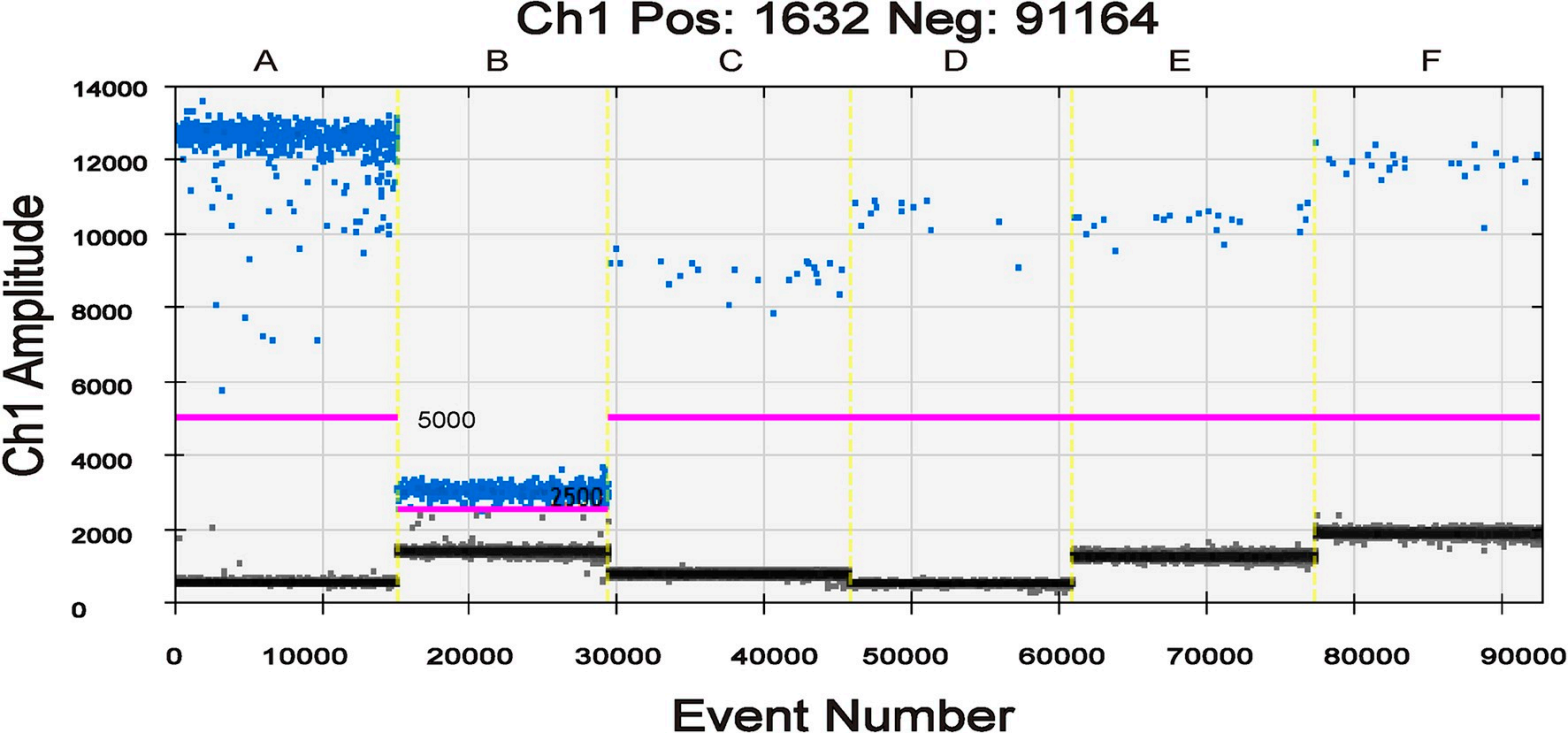
The ddPCR results for DNA targets in the same samples. The horizontal axis indicates the event number of six DNA targets and the vertical axis indicates sample amplitude. The positive and negative droplets as classified by Thresholds (pink lines) of individual wells are shown in blue and grey, respectively. Thresholds of *RLEP* (A), *esxA* (C), *pra* (D), *HSP18* (E) and *Ag85* (F) for positive detection are set to 5,000, and Threshold of *groEL* (B) is set to 2,500. The different amplitudes of positive droplets were observed when different DNA targets were applied (*RLEP*, Pos: 782, Neg: 14,436; *groEL*, Pos: 769, Neg: 13,536; *esxA*, Pos: 22, Neg: 16,386; *pra*, Pos: 12, Neg: 15,029; *HSP18*, Pos: 21, Neg: 16,494; *Ag85B*, Pos: 26, Neg: 15,283). The total positive and negative droplets of six DNA targets are 1,632 and 91,164, respectively.

The results of the sensitivities of 17 target genes detected by qPCR in five MB patients are shown in S2 Table. *RLEP*, *groEL*, proline-rich antigen (*pra*), *esxA*, *HSP18* and *85B* target genes showed a higher sensitivity than other genes, of which LOD was lower than 1:2,000 (0.5 copies/ul). Among the six target genes, the two most sensitive genes were *RLEP* and *groEL*, given that the CT values of *RLEP* and *groEL* were less than 38 at the dilution ratios of 1:2,000. Moreover, *RLEP* and *groEL* showed more than 10 times the sensitivity of the other four genes in ddPCR (Fig 1).

We further evaluated the specificity of *RLEP* and *groEL* genes. Neither *Mycobacterium tuberculosis* nor *M*. *marinum* yielded positive results by qPCR or ddPCR. Therefore, *RLEP* and *groEL* were finally selected as the DNA targets to establish the ddPCR assay.

### Determination of the cut-off of duplex ddPCR assay in leprosy diagnosis

Skin biopsies from 59 non-leprosy patients that were diagnosed as inflammatory diseases, such as psoriasis, lichen planus, served as negative controls to define the cut-off of the duplex ddPCR assay. The mean positive events for *RLEP* were 0.34±0.56 (95% CI 0.19–0.49) and the maximum value was two. For *groEL*, the mean positive events were 0.49±0.67 (95% CI 0.32–0.67) with a maximum score of three (S3 Table). A positive result of ddPCR assay was determined as follows: 1) the threshold line for *RLEP* and *groEL* was 5,000 and 2,500, respectively (Fig 1); 2) the well was marked as a positive well if more than four fluorescent signal events were shown above the threshold line to avoid false positive; and 3) the sample, which was present in at least three positive wells, was defined as an *M*. *leprae*-infected sample.

### The comparison of duplex ddPCR with qPCR in leprosy diagnosis

Of the 68 MB patients, the sensitivity of qPCR and ddPCR were both 100%. No case of non-leprosy patients showed positive results in both qPCR and ddPCR assays, showing a specificity of 100%. Out of 44 PB patients, qPCR was positive in 16 patients (36.4%; 95% confidence interval [CI], 23.7 to 51.2%). In contrast, ddPCR detected *M*. *leprae* in 35 patients (79.5%; 95% CI, 65.3 to 89.1%). A total of 16 patients (36.4%; 95% CI, 23.7 to 51.2%) tested positively by both qPCR and ddPCR. There was no case in which qPCR was positive and ddPCR was negative. The ddPCR confirmed the diagnosis in 19 out of 28 skin tissues (67.9%; 95% CI, 49.2 to 82.2%) which were qPCR negative (Table 2). Comparative analysis of the positivity between qPCR and ddPCR indicated that the sensitivity of ddPCR was significantly higher than that of qPCR in our study (P<0.001).

**Table 2:** Performance characteristic of qPCR and ddPCR for the detection of *M*. *leprae* in leprosy patients.

	qPCR			ddPCR		
Number of patients	MB	PB	Non-leprosy	MB	PB	Non-leprosy
**Positive**	68	16	0	68	35	0
**Negative**	0	28	59	0	9	59
**Total**	68	44	59	68	44	59
**Performance characteristic (**percent (95% CI))						
**Sensitivity**	100 (93.6–100)	36.4 (23.7–51.2)		100 (93.6–100)	79.5 (65.3–89.1)	
**Specificity**	100 (92.7–100)	100 (92.7–100)		100 (92.7–100)	100 (92.7–100)	
**PPV**^a^	100 (93.6–100)	100 (77.3–100)		100 (93.6–100)	100 (88.2–100)	
**NPV**^b^	100 (92.7–100)	67.8 (57.4–76.7)		100 (92.7–100)	86.8 (76.5–93.1)	
**-LR**^c^	0	63.6 (48.8–76.3)		0	20.4 (10.9–34.7)	
**Accuracy**	100 (96.6–100)	72.8 (63.5–80.5)		100 (96.6–100)	91.3 (84.0–95.5)	

^a^PPV = positive predictive value.

^b^NPV = negative predictive value.

^c^-LR = negative likelihood ratio.

## Discussion

We developed a duplex ddPCR assay for leprosy diagnosis in skin biopsies that performed with increased sensitivity compared to qPCR, particularly for PB patients. To the best of our knowledge, this is the first systematic comparative evaluation between ddPCR and qPCR for the detection of *M*. *leprae* DNA.

In the current study, we systematically compared the sensitivity of 17 target genes for detecting *M*. *leprae*, and confirmed *RLEP* and *groEL* were the most sensitive genes. We firstly employed 17 target genes from previous studies [13,15–26]. The specificity and sensitivity assays showed that *RLEP* was the optimal gene, similar to results from previous studies [7,15,16], followed by *groEL*. Therefore, this ddPCR assay accurately detected *M*. *leprae* using *RLEP* and *groEL*, due to the fact that the duplex ddPCR assay increased throughput compared to a singleplex method used in previous studies [27,28]. *RLEP* is *M*. *leprae*-specific repetitive element and one of the most frequent genes used with a high sensitivity for detecting *M*. *leprae* [7,15,16]. Martinez et al reported that the sensitivity of *RLEP* was highest as compared to other three target genes (*soda*, *85B* and *16SrRNA*), which coincides with our findings [14]. *groEL* gene encoding the 65-kD GroEL antigen in the cell wall of *M*. *leprae* also showed good sensitivity in detecting *M*. *leprae* DNA [20], and in this study its sensitivity was similar to *RLEP*. While other genes (*16SrRNA*, *esxA*, *Ag85B*, *sodA*, *pra*, *rpoT*, *ML2179*, *ML1545*, *ML0098*, *ML0024*, *MntH*, *AT repeats*, *AGT repeats*, *TTC repeats*) with the sensitivity ranged from 20% to 94% in previous studies.[13–22,24–26,29] showed decreased sensitivities compared to *RLEP* and *groEL* in this study.

DNA normalization is one of the critical steps in establishment of PCR-based methods. The DNA samples from five MB patients were normalized and gradient diluted to assess the analytical sensitivity of all DNA targets for detecting *M*. *leprae*. Although some researchers have favored the purified pathogen for comparative evaluation [30,31], normalized test samples are more appropriate for practical applications and are more easily accessible.

The suitable classification of positive and negative droplets play an important role in the readout of ddPCR. We demonstrated that unlike *RLEP*, the partitions of the *groEL* gene were not suitable for automated threshold assignment because the difference in fluorescence intensity between positive and negative events was not apparent. Determining the correct threshold line for *groEL* is needed as the generated droplets are identified as positive or negative based on a threshold at a certain fluorescence level. Our manual threshold line was defined as the mean fluorescence signal in the clinical samples diagnosed as non-leprosy plus a number of standard deviations according to the clustering method developed by Jones *et al* [32] and the single threshold determination method proposed by Dreo *et al* [33]. Finally, a reliable criterion in the developed duplex ddPCR assay was determined by 59 controls. This threshold was in agreement to some extent with previous work demonstrating that one out of three wells of negative controls with no template had two or three positive droplets for HIV-1 RNA assay described by Kiselinova *et al* [34].

As expected, qPCR and ddPCR turned out to be the same 100% sensitivity for bacteriological confirmation on MB patients. While our study clearly revealed that the duplex ddPCR assay was more sensitive than qPCR in diagnosing PB leprosy by analyzing 44 PB samples (sensitivity: 36.4% vs 79.5% in qPCR and ddPCR, respectively). Our data are consistent with previous studies comparing ddPCR and qPCR [8–11]. The sensitivity of qPCR and ddPCR was considered to be comparable in other study designed for the enumeration of *Cryptosporidium oocysts* [35] while ddPCR outperformed SYBR green-based qPCR for the detection of *fecal enterotoxigenic Bacteroides fragilis* [36]. Leprosy as a complex disease is common to observe a very long incubation period to leprosy outcome and subclinical stages with dormant *M*. *leprae* within granulomas are likely to occur [1]. As shown in a previous study [11], some patients clinically and histologically classified as non-leprosy did in fact have leprosy by qPCR. Therefore, duplex ddPCR may be a better method to improve clinical management decisions on leprosy diagnosis, especially for difficult-to-diagnose patients.

We acknowledge that this study has some limitations. Firstly, as this study served as a preliminary exploration, advanced studies are needed with a higher number of PB samples to confirm these findings. And also ddPCR is costly and rather cumbersome and technically demanding, which lead it difficult to transfer to the routine clinical situation. Further work on the simplification of this test is necessary. Moreover, threshold settings remain a challenging but crucial task, because the current generation of ddPCR is not fitted with a fluorescence intensity sorter to allow for target confirmation by sequencing. More clinical practice is needed to refine the standard of duplex ddPCR assay for leprosy diagnosis.

In summary, the duplex ddPCR assay, targeting *RLEP* and *groEL*, provided a high sensitive method for the diagnosis of PB leprosy. Furthermore, ddPCR will be a valuable technology and with additional improvements in prospect, it is likely to mature into an indispensable tool in future clinical and basic research of leprosy.

### Accession numbers

*RLEP*: NC_002677.1 (39269.39991). *groEL*: Gene ID: 908906. *pra*: Gene ID: 908610. *esxA*: Gene ID: 908212. *HSP18*: Gene ID: 910696. *85B*: Gene ID: 909036. *rpoT*: Gene ID: 910077. *ML0024*: Gene ID: 909040. *ML1545*: Gene ID: 909602. *ML2179*: Gene ID: 908978. *soda*: Gene ID: 910514. *16SrRNA*: Gene ID: 910245. *TTC*: Gene ID: 908674/908673. *ML0098*: Gene ID: 908293. *AT*: Gene ID: 909755/909757. *MntH*: Gene ID: 908932. *AGT*: Gene ID: 908866/908865.

## Supporting information

S1 Table Sequences of primers and probes for qPCR and ddPCR. (PDF)

S2 Table Ct values of all target genes in serial dilutions of 5 MB clinical samples. The sensitivities of 17 target genes were assessed by qPCR using a series of normalized DNA samples from MB patients.
(PDF)

S3 Table The mean positive events of 59 negative controls in the duplex ddPCR assay. Skin biopsies from 59 dermatological patients that were not leprosy served as negative controls used to define the cut-off of the duplex ddPCR assay.
(PDF)

S1 File STARD checklist. (PDF)

S2 File STARD flow diagram: ddPCR. (PDF)

S3 File STARD flow diagram: qPCR. (PDF)
