# Supplementary material for: Development and evaluation of a droplet digital PCR assay for the diagnosis of paucibacillary leprosy in skin biopsy specimens
Source: PLoS Negl Trop Dis. 2019 Mar 18;13(3):e0007284. doi: 10.1371/journal.pntd.0007284 (PMC6438576; doi:10.1371/journal.pntd.0007284)
Supplement: S1 Table — (PDF) [file pntd.0007284.s001.pdf]

S1 Table. Sequences of primers and probes for qPCR and ddPCR.

Name	Oligo sequences 5' to 3'			Product Size (bp)	TM	References
	Forward primer	Reverse primer	Taqman probe			
**RLEP**	GCAGCAGTATCGTGTTAGTGAA	CGCTAGAAGGTTGCCGTAT	FAM-CGCCGACGGCCGGATCATCGA-MGB	73	60	[1,2]
**HSP18**	CGATCGGGAAATGCTTGC	CGAGAACCAGCTGACGATTG	FAM-ACACCGCGTGGCCGCTCG-MGB	62	62	[3]
**16SrRNA**	GCATGTCTTGTGGTGGAAAGC	CACCCCACCAACAAGCTGAT	FAM-CATCCTGCACCGCA-MGB	71	71	[2]
**esxA**	CGTTTCAGCCGAGTGATTGA	CCGAGGGAATAAACCATGCA	FAM-TGCTTGCACCAGGTCGCCCA-MGB	62	62	[3]
**Ag85B**	GTGCACGGCAGCAACCTAA	TCGGCATTGAGGTTGAACAC	FAM-TTCCAGGACGCCTACAACGGTGCT-MGB	80	60	[4]
**sodA**	CACCCTGCCCGATTTGG	CTTGGTGTGGTGGATCTCGTT	FAM-ACTACGCAGCGCTGGAACCACATATCT-MGB	82	60	[2]
**pra**	CACACCGTCGTCATGATCTTG	CCGCTGTGGGACTCCAAA	FAM-CGCCAGGGTTTGC-MGB	54	60	[5,6]
**groEL**	GCCGGGTGCAGCAGTATC	CCGACGGCCGGATCA	FAM-TGTTAGTGAACAGTGCATCG-MGB	63	60	[7]
**rpoT**	TGACTACGTCGTCGTCTTCGA	TGAGCCCAGCGAGGACAT	FAM-AGACCGAGGTCGGC-MGB	80	60	[8]
**ML2179**	ACGCTCGCGTATGTCAAAAA	TCAACCTAGCCAATTTATCGATGA	FAM-ATTTTGACCCATCGTTATAT-MGB	68	58	[9]
**ML1545**	CTCCGTCCTCCGTCTTGCT	GGCTCCCTTGGGATGGTTT	FAM-ACTGTGAAATAGTTCATGCGG-MGB	61	60	[9,10]
**ML0098**	TCGCTGCGGCCGTACTAC	TGAAAGCGACCGGAATATCC	FAM-AACCGCTGGCAACG-MGB	111	60	[4]
**ML0024**	GGTGCACGATCACGATGGT	TCCGGTTTGTCGTCGAAAG	FAM-TCGATTCCCTGGCAGAT-MGB	57	60	[11]
**MntH**	ACCCGCTTGGACGTCATC	TCGCCGTATTCACGATTCC	FAM-TGGCGATGACGATCG-MGB	55	58	[12]
**AT repeats**	CGGGTTGGCGCTTCTG	CGCCCATCACCCTAGCTTT	FAM-TATGGCTGCGCAGTTG-MGB	57	60	[12,13]
**AGT repeats**	TCAACGCTGCGGTTTCG	CCTTGGCAGGCGGTGAT	FAM-TAGTCGCGCAGATGC-MGB	59	58	[12,13]
**TTC repeats**	CGGGAATAAGTTTAGCATTGAAGAA	TCCGTTGGGTTCGATCGA	FAM-AGAACTCGCCGATGTC-MGB	124	60	[12,13]

References:

1. Moher D, Liberati A, Tetzlaff J, Altman DG, Group P. Preferred reporting items for systematic reviews and meta-analyses: the PRISMA statement. BMJ. 2009;339: b2535. doi: 10.1136/bmj.b2535.
2. Yan W, Xing Y, Yuan LC, De Yang R, Tan FY, Zhang Y, et al. Application of RLEP real-time PCR for detection of M. leprae DNA in paraffin-embedded skin biopsy specimens for diagnosis of paucibacillary leprosy. Am J Trop Med Hyg. 2014;90(3): 524-529. doi: 10.4269/ajtmh.13-0659.
3. Davis GL, Ray NA, Lahiri R, Gillis TP, Krahenbuhl JL, Williams DL, et al. Molecular assays for determining Mycobacterium leprae viability in tissues of experimentally infected mice. PLoS Negl Trop Dis. 2013;7(8): e2404. doi: 10.1371/journal.pntd.0002404.
4. Martinez AN, Britto CF, Nery JA, Sampaio EP, Jardim MR, Sarno EN, et al. Evaluation of real-time and conventional PCR targeting complex 85 genes for detection of Mycobacterium leprae DNA in skin biopsy samples from patients diagnosed with leprosy. J Clin Microbiol. 2006;44(9): 3154-3159. doi: 10.1128/JCM.02250-05.
5. Caleffi KR, Hirata RD, Hirata MH, Caleffi ER, Siqueira VL, Cardoso RF. Use of the polymerase chain reaction to detect Mycobacterium leprae in urine. Braz J Med Biol Res. 2012;45(2): 153-157.
6. Arunagiri K, Sangeetha G, Sugashini PK, Balaraman S, Showkath Ali MK. Nasal PCR assay for the detection of Mycobacterium leprae pra gene to study subclinical infection in a community. Microb Pathog. 2017;104: 336-339. doi: 10.1016/j.micpath.2017.01.046.
7. Qinxue W, Xinyu L, Wei H, Tao L, Yaoping Y, Jinping Z, et al. A study on PCR for detecting infection with M. leprae. Chin Med Sci J. 1999;14(4): 237-241.
8. Turankar RP, Pandey S, Lavania M, Singh I, Nigam A, Darlong J, et al. Comparative evaluation of PCR amplification of RLEP, 16S rRNA, rpoT and Sod A gene targets for detection of M. leprae DNA from clinical and environmental samples. Int J Mycobacteriol. 2015;4(1): 54-59. doi: 10.1016/j.ijmyco.2014.11.062.
9. Chaitanya VS, Cuello L, Das M, Sudharsan A, Ganesan P, Kanmani K, et al. Analysis of a novel multiplex polymerase chain reaction assay as a sensitive tool for the diagnosis of indeterminate and tuberculoid forms of leprosy. Int J Mycobacteriol. 2017;6(1): 1-8. doi: 10.4103/2212-5531.201885.
10. Sundeep Chaitanya V, Das M, Eisenbach TL, Amoako A, Rajan L, Horo I, et al. Mycobacterium leprae specific genomic target in the promoter region of probable 4-alpha-glucanotransferase (ML1545) gene with potential sensitivity for polymerase chain reaction based diagnosis of leprosy. Int J Mycobacteriol. 2016;5(2): 135-141. doi: 10.1016/j.ijmyco.2016.01.002.

11. da Silva Martinez T, Nahas AA, Figueira MM, Costa AV, Goncalves MA, Goulart LR, et al. Oral lesion in leprosy: borderline tuberculoid diagnosis based on detection of Mycobacterium leprae DNA by qPCR. Acta Derm Venereol. 2011;91(6): 704-707. doi: 10.2340/00015555-1175.
12. Cruz AF, Furini RB, Roselino AM. Comparison between microsatellites and Ml MntH gene as targets to identify Mycobacterium leprae by PCR in leprosy. An Bras Dermatol. 2011;86(4): 651-656.
13. Young SK, Taylor GM, Jain S, Suneetha LM, Suneetha S, Lockwood DN, et al. Microsatellite mapping of Mycobacterium leprae populations in infected humans. J Clin Microbiol. 2004;42(11): 4931-4936. doi: 10.1128/JCM.42.11.4931-4936.2004.
