# Supplementary material for: Development and evaluation of a droplet digital PCR assay for the diagnosis of paucibacillary leprosy in skin biopsy specimens
Source: PLoS Negl Trop Dis. 2019 Mar 18;13(3):e0007284. doi: 10.1371/journal.pntd.0007284 (PMC6438576; doi:10.1371/journal.pntd.0007284)
Supplement: S2 Table — The sensitivities of 17 target genes were assessed by qPCR using a series of normalized DNA samples from MB patients. (PDF) [file pntd.0007284.s002.pdf]

**S2 Table. Ct values of all target genes in serial dilutions of 5 MB clinical samples.**

| Target gene | 1:10 | | 1:100 | | 1:200 | | 1:1000 | | 1:2000 | | 1:10000 | |
|---|---|---|---|---|---|---|---|---|---|---|---|---|
| | Mean | CV[a] | Mean | CV | Mean | CV | Mean | CV | Mean | CV | Mean | CV |
| **RLEP** | 26.12 | 0.01 | 29.58 | 0.01 | 30.71 | 0.02 | 33.43 | 0.02 | 34.48 | 0.01 | 36.75 | 0.02 |
| **groEL** | 29.06 | 0.01 | 32.82 | 0.01 | 33.65 | 0.01 | 36.22 | 0.01 | 37.59 | 0.01 | ND[b] | - |
| **pra** | 30.97 | 0.01 | 34.61 | 0.01 | 35.40 | 0.01 | 38.29 | 0.01 | 38.45 | 0.01 | ND | - |
| **esxA** | 31.27 | 0.01 | 34.80 | 0.01 | 35.93 | 0.01 | 38.25 | 0.01 | 38.39 | 0.01 | ND | - |
| **HSP18** | 31.58 | 0.01 | 35.07 | 0.01 | 36.23 | 0.02 | 38.94 | 0.01 | 39.14 | 0.01 | ND | - |
| **85B** | 32.07 | 0.01 | 35.67 | 0.01 | 36.86 | 0.02 | 38.98 | 0.01 | 39.42 | 0.00 | ND | - |
| **rpoT** | 32.17 | 0.01 | 35.76 | 0.01 | 36.61 | 0.02 | 39.01 | 0.01 | ND | - | ND | - |
| **ML0024** | 32.29 | 0.01 | 35.93 | 0.01 | 37.07 | 0.01 | 38.66 | 0.00 | ND | - | ND | - |
| **ML1545** | 32.42 | 0.01 | 36.07 | 0.01 | 37.08 | 0.01 | 39.09 | 0.01 | ND | - | ND | - |
| **ML2179** | 32.61 | 0.01 | 36.23 | 0.01 | 37.63 | 0.01 | 39.28 | 0.01 | ND | - | ND | - |
| **sodA** | 33.08 | 0.01 | 36.85 | 0.01 | 38.00 | 0.01 | 39.37 | 0.01 | ND | - | ND | - |
| **16SrRNA** | 33.09 | 0.01 | 36.94 | 0.01 | 37.72 | 0.01 | ND | - | ND | - | ND | - |
| **TTC** | 33.37 | 0.01 | 37.41 | 0.02 | 38.29 | 0.01 | ND | - | ND | - | ND | - |
| **ML0098** | 33.42 | 0.01 | 37.02 | 0.01 | 38.03 | 0.02 | ND | - | ND | - | ND | - |
| **AT** | 33.49 | 0.01 | 37.21 | 0.01 | 38.16 | 0.01 | ND | - | ND | - | ND | - |
| **MntH** | 37.35 | 0.01 | ND | - | ND | - | ND | - | ND | - | ND | - |
| **AGT** | ND | - | ND | - | ND | - | ND | - | ND | - | ND | - |

The sensitivities of 17 target genes were assessed by qPCR using a series of normalized DNA samples from MB patients.

[a]CV = Coefficient of variation.

[b]ND = Not detected.
