# Supplementary material for: Development and evaluation of a droplet digital PCR assay for the diagnosis of paucibacillary leprosy in skin biopsy specimens
Source: PLoS Negl Trop Dis. 2019 Mar 18;13(3):e0007284. doi: 10.1371/journal.pntd.0007284 (PMC6438576; doi:10.1371/journal.pntd.0007284)
Supplement: S3 Table — Skin biopsies from 59 dermatological patients that were not leprosy served as negative controls used to define the cut-off of the duplex ddPCR assay. (PDF) [file pntd.0007284.s003.pdf]

S3 Table. No. of possitive events for 59 control cases.

Number	RLEP			groEL		
	1	2	Mean	1	2	Mean
**1**	0	0	0	0	0	0
**2**	0	1	0.5	0	1	0.5
**3**	1	0	0.5	0	0	0
**4**	2	0	1	0	1	0.5
**5**	0	1	0.5	0	0	0
**6**	0	2	1	0	0	0
**7**	2	1	1.5	0	1	0.5
**8**	0	0	0	0	0	0
**9**	1	2	1.5	0	1	0.5
**10**	0	1	0.5	3	3	3
**11**	2	0	1	4	1	2.5
**12**	0	1	0.5	1	0	0.5
**13**	2	0	1	1	1	1
**14**	0	0	0	0	0	0
**15**	0	0	0	1	0	0.5
**16**	0	0	0	0	1	0.5
**17**	0	0	0	1	0	0.5
**18**	0	0	0	1	1	1
**19**	1	0	0.5	2	1	1.5
**20**	0	0	0	0	1	0.5
**21**	0	0	0	0	0	0
**22**	0	0	0	0	0	0
**23**	0	0	0	1	0	0.5
**24**	0	0	0	0	0	0
**25**	0	0	0	1	0	0.5
**26**	0	0	0	1	2	1.5
**27**	0	0	0	1	1	1
**28**	1	0	0.5	0	0	0
**29**	0	0	0	0	0	0
**30**	0	0	0	0	0	0
**31**	0	0	0	0	0	0
**32**	0	0	0	1	1	1
**33**	0	0	0	0	0	0
**34**	0	0	0	0	1	0.5
**35**	0	0	0	0	1	0.5
**36**	0	0	0	0	0	0
**37**	0	0	0	0	1	0.5
**38**	0	0	0	0	1	0.5
**39**	0	0	0	0	0	0
**40**	0	0	0	1	0	0.5
**41**	0	0	0	0	1	0.5

**42**	0	0	0	0	0	0
**43**	0	0	0	0	0	0
**44**	0	0	0	0	1	0.5
**45**	0	0	0	1	0	0.5
**46**	0	0	0	2	1	1.5
**47**	0	3	1.5	0	0	0
**48**	0	0	0	4	1	2.5
**49**	0	0	0	2	1	1.5
**50**	0	0	0	0	0	0
**51**	1	0	0.5	0	1	0.5
**52**	0	0	0	0	0	0
**53**	0	0	0	0	0	0
**54**	1	2	1.5	0	0	0
**55**	2	0	1	0	0	0
**56**	4	0	2	0	0	0
**57**	2	0	1	0	0	0
**58**	0	4	2	1	0	0.5
**59**	0	0	0	0	1	0.5
