# Supplementary figures and images for: Development and evaluation of a droplet digital PCR assay for the diagnosis of paucibacillary leprosy in skin biopsy specimens
Source: PLoS Negl Trop Dis. 2019 Mar 18;13(3):e0007284. doi: 10.1371/journal.pntd.0007284 (PMC6438576; doi:10.1371/journal.pntd.0007284)

**STARD flow diagram: ddPCR**

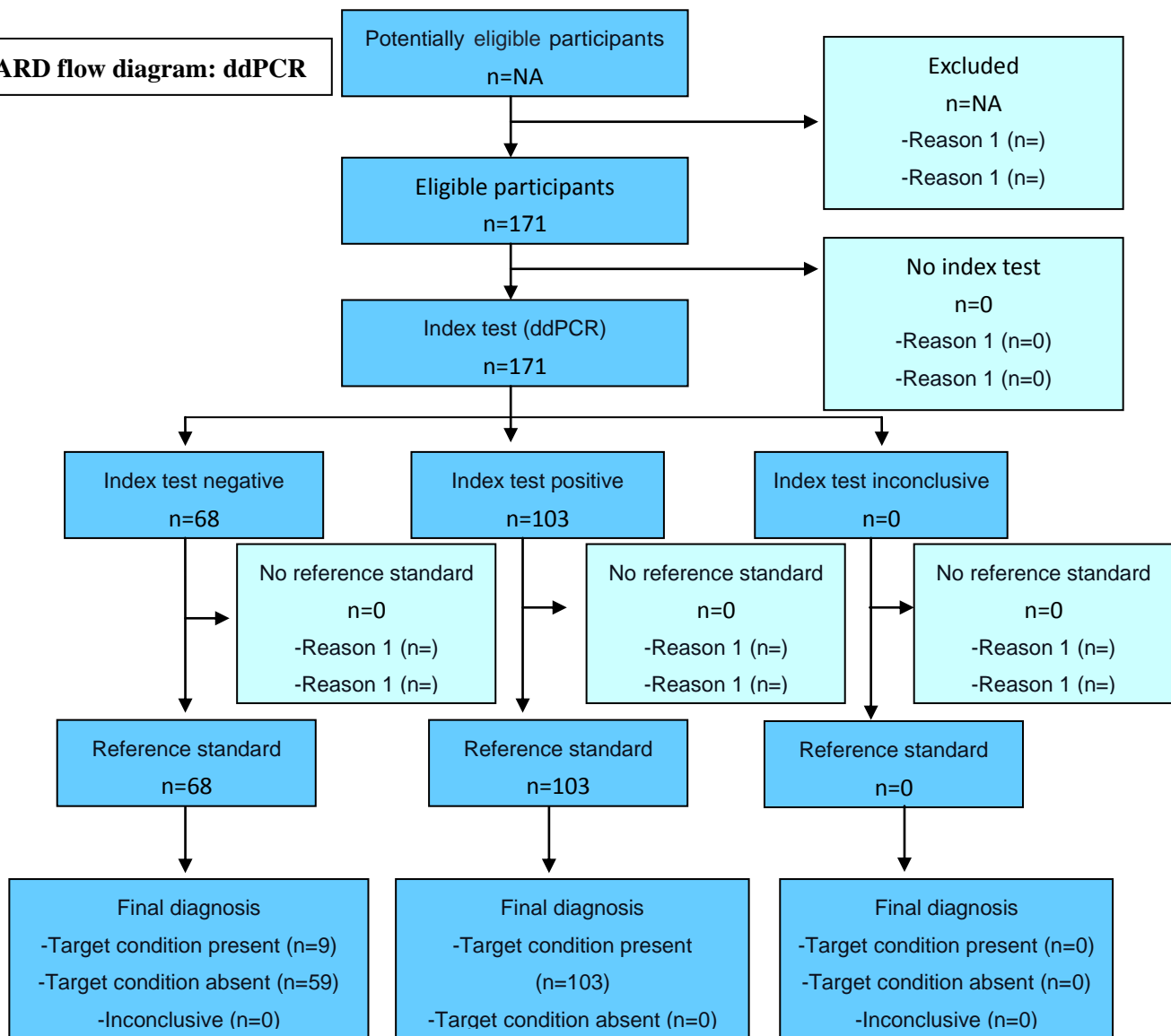

Supplement: S2 File — (PDF) [file pntd.0007284.s005.pdf]

STARD flow diagram: qPCR

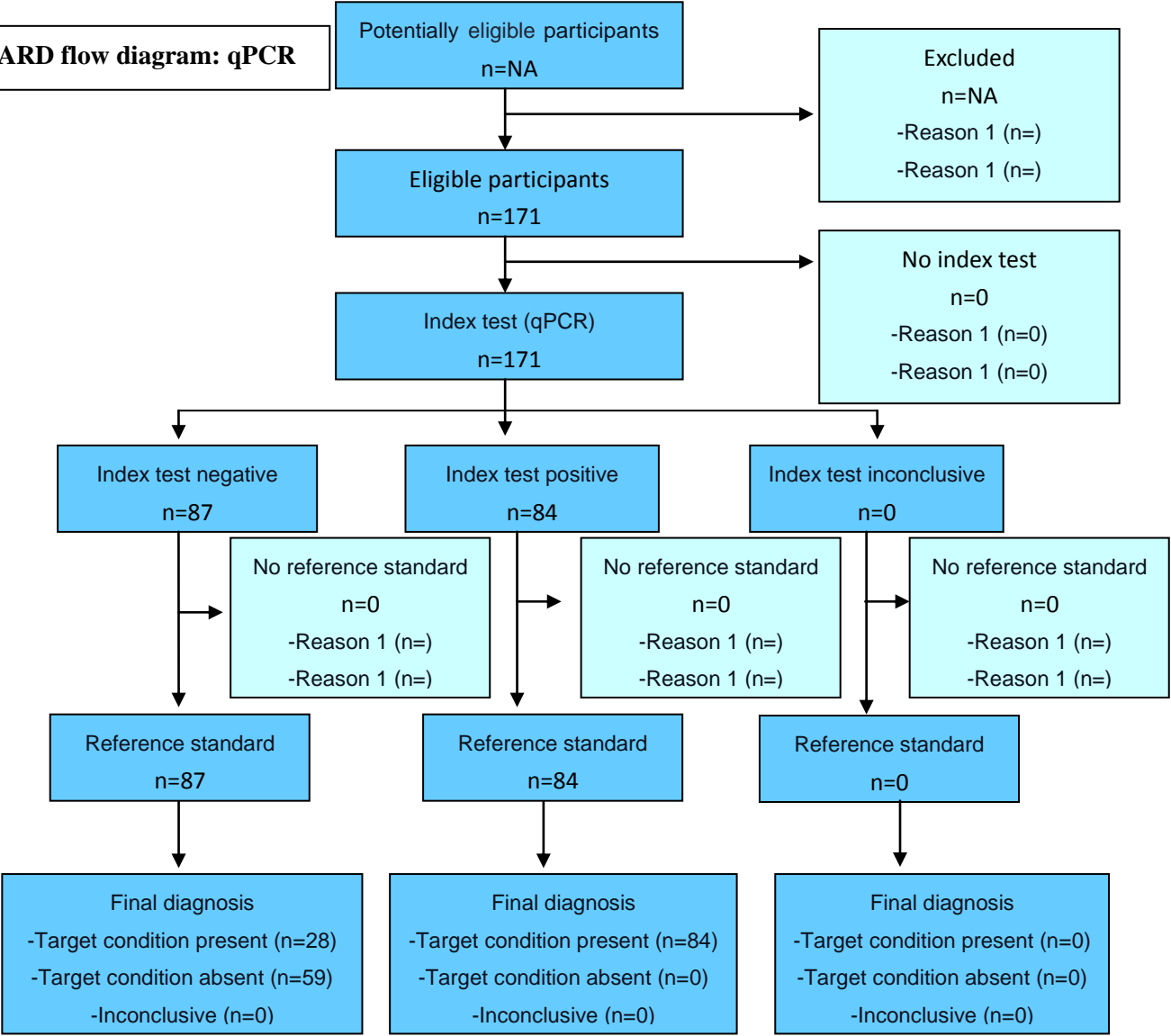

Supplement: S3 File — (PDF) [file pntd.0007284.s006.pdf]
